# Improving the Physical Properties of Starch-Based Powders for Potential Anti-Adhesion Applications

**DOI:** 10.3390/polym15244702

**Published:** 2023-12-13

**Authors:** Jaydon Sun, Tzu-Shan Fang, Yu-Xiang Chen, Yu-Cheng Tsai, Yi-Xin Liu, Chih-Yu Chen, Chen-Ying Su, Hsu-Wei Fang

**Affiliations:** 1 Thomas Jefferson High School for Science and Technology, Alexandria, VA 22312, USA; jaydonsun24@gmail.com; 2 High-Value Biomaterials Research and Commercialization Center, National Taipei University of Technology, Taipei 10608, Taiwan; clairefang06@gmail.com (T.-S.F.); a0968709781@gmail.com (Y.-X.C.); yuchen898989@gmail.com (Y.-C.T.); dr.evieliu@gmail.com (Y.-X.L.); 3 Taipei WEGO Private Senior High School, Taipei 11254, Taiwan; 4 Department of Chemical Engineering and Biotechnology, National Taipei University of Technology, Taipei 10608, Taiwan; 5 Department of Orthopedics, Shuang Ho Hospital, Taipei Medical University, New Taipei City 23561, Taiwan; aleckc2424@gmail.com; 6 Department of Orthopedics, School of Medicine, College of Medicine, Taipei Medical University, Taipei 11042, Taiwan; 7 Institute of Biomedical Engineering and Nanomedicine, National Health Research Institutes, Miaoli 35053, Taiwan

**Keywords:** starch, water absorption, viscosity, salt, anti-adhesion

## Abstract

Postoperative adhesion is one of the most common complications that occur during and after surgery; thus, materials that can prevent adhesion are often applied. Starch powders with a high water absorption capacity are preferred, and many studies have focused on increasing the water absorption of modified starches, as native starch powders display poor water-holding capacities. The effects of salts on the physical properties of acetylated distarch phosphate potato starch powders were investigated here. Changes in functional groups, the crystal structures of modified starch, particle morphologies, water absorption, viscosity, and in vivo adhesion were investigated. The results showed that salts greatly improved the water absorption and viscosity of acetylated distarch phosphate potato starch powders. Among the three different salt-modified starch powders, NaCl-modified starch powders displayed higher water absorption and viscosity and demonstrated better in vivo anti-adhesion performance. The results of this study propose a potential biomaterial that may function as an anti-adhesive, potentially leading to reduced surgical risks and a better quality of life for patients.

## 1. Introduction

Although advances in surgical techniques and pharmaceutical therapies have improved surgical quality, postoperative adhesions are still one of the most common complications that affect patients’ quality of life [1,2]. Adhesions are the formation of tissues that connect otherwise separate body surfaces, and unlike many other postoperative complications, they have lifelong effects, some of which include bowel obstruction, secondary infertility, and pelvic pain [3,4]. Excessive blood loss is also another major clinical complication during surgery, resulting in an increase in morbidity and mortality [5]. Out of 506 patients undergoing a surgical procedure, the average amount of blood loss was 273.23 milliliters (mL), which is around 5% of the total amount of blood in a human body [6]. Therefore, materials that can function as hemostasis or anti-adhesion materials are often applied.

Polysaccharides are naturally hydrophilic and hygroscopic and are generally biodegradable, nontoxic, and biocompatible, and thus they are widely applied for hemostasis, wound dressing, drug delivery, adhesion prevention, tissue engineering scaffolds, etc. [7,8,9,10]. To stop bleeding, polysaccharides such as chitosan, cellulose, starch, or alginate may adsorb positively charged platelets, activate clotting factors, absorb water rapidly, or rapidly form glue as a clotting mechanism [11]. For wound dressing, polysaccharide-based hydrogels are often used. As wound dressings require excellent tissue adhesion, swelling, water absorption, and other functions such as anti-inflammation, polysaccharide-based hydrogels usually undergo some physical or chemical modifications or are combined with other materials [12]. It has been shown that chitosan/gelatin/polyvinyl alcohol (PVA) hydrogels greatly improve tensile strength and display good hemostasis, swelling ability, and water evaporation, resulting in a promising material for wound dressing [13]. Moreover, chitosan/gelatin/PVA or chitosan/PVA hydrogels demonstrate good antibacterial activity when optimal concentrations of honey are added, resulting in accelerated wound healing [14,15]. For anti-adhesion, starch is a favorable option due to its high hydrophilicity out of all the polysaccharides [16]. One current starch-based powder product on the market, 4DryField^®^ (PlantTec Medical, Luneburg, Germany), is made using potato starches that are applied as hemostatic agents and anti-adhesive materials. The hemostatic mechanism achieved by using 4DryField^®^ is to absorb water from the blood, resulting in the concentration of coagulation factors and the acceleration of hemostasis [17]. In addition, saline solution can be added onto 4DryField^®^ after hemostasis to form a gel, forming a physical barrier that can prevent adhesion [18]. Therefore, the water absorption capacity of polysaccharide-based hydrogels or starch-based powders is critical for acting as hemostatic, wound dressing, or anti-adhesive materials.

Natural starches exhibit low water absorption capabilities and swelling power; thus, modification is required to improve their functional properties [19]. The modification of starches can be achieved using physical, chemical, biological, or combined methods [20]. Depending on the source of the native starch, different modification methods can be applied to improve its water absorption ability. Regarding physical modifications, the drum drying of pregelatinized sweet potato, twin-screw extrusion of corn and potato starch, and ball milling of water caltrop and horseshoe starch have been shown to increase the water absorption capacities of starch [21,22,23]. Regarding chemical modifications, the oxidation or acetylation of various starches has been shown to enhance their water absorption capabilities [24,25,26,27]. The main goal of all these modifications is either to change the interactions between the water and starch molecules (physical modification) or to add some essential functional groups onto the starch molecules due to the presence of abundant hydroxyl groups in the starch, resulting in an increase in its water absorption capacity [28]. In addition, salts are often added to starch in the food industry to delay gelatinization or inhibit retrogradation, resulting in a longer shelf life without changing the quality of the starch food. The purpose of adding salts to starch is to increase its water absorption capacity; thus, the final product can contain more than 75% water, which has been shown to effectively reduce retrogradation [29]. A similar concept has been applied to porous starch loaded with crystallized calcium ions, which greatly improves its hemostatic function [30]. However, whether starch loaded with salts can absorb more water to act as an anti-adhesion agent has not yet been investigated extensively.

In this study, the effect of salts on the water absorption ability of acetylated distarch phosphate (modified potato starch) was investigated. By using emulsification, different salts were incorporated into acetylated oxidized potato starch, and the powder’s morphology was observed. The functional groups and internal atomic structures of starch powders were analyzed. Both the water absorption and viscosity of different salt-modified starch powders were investigated. In addition, different salt-modified starch powders were applied in vivo to investigate potential anti-adhesion applications.

## 2. Materials and Methods

### 2.1. Modification of Starch-Based Powders

Modifying the starch consisted of 2 phases: a water phase and an oil phase. For the water phase, acetylated distarch phosphate (molecular weight is 990.84 g/mol, Gemfont Corporation, Taipei, Taiwan), deionized water, sodium chloride (NaCl, J.T.Baker^®^, Phillipsburg, NJ, USA), calcium chloride (CaCl_2_, Acros Organics, Waltham, MA, USA), and potassium chloride (KCl, Sigma-Aldrich, St. Louis, MO, USA) were required. The oil phase consisted of span 80 (Emperor Chemical Co., Ltd., Xiao Shan City, China), tween 80 (Emperor Chemical Co., Ltd.), and sunflower oil (Standard Foods, Taipei, Taiwan).

First, 3 g of NaCl or CaCl_2_ or KCl was added to 25 milliliters (mL) of deionized water and stirred until completely dissolved. Two grams of acetylated distarch phosphate powder was then added to the salt solution and were stirred at 700 revolutions per minute (rpm) for 1 h until the hydrogel was formed. To convert the hydrogel into spherical particles with uniform particle size distributions, the water-in-oil emulsification strategy was used. Span 80 and tween 80 were added to sunflower oil as the oil phase when the final ratios were 2.25% and 0.25% (*v*/*v*), respectively. The oil phase was stirred at 1000 rpm in a water bath at 80 °C. Subsequently, the hydrogel was added to the oil phase while stirring vigorously. After 30 min, the mixture was cooled to room temperature and removed from the stir. The mixture was then centrifuged at 5000 rpm to separate the oil from the aqueous phase. After removing the oil, the solution was washed with 99% ethanol 3 times. Finally, the solution with modified starch particles was filtered and dried for 30 min to obtain the modified powders. The powder was ground and sifted until the size was less than 0.125 mm.

### 2.2. Fourier-Transform Infrared Spectroscopy and X-ray Diffractometer Analysis

A total of 0.2 g of starch powders was used to evaluate and identify the functional groups, and the Fourier-Transform Infrared (FTIR, Perkin Elmer, Spotlight 200i Sp2 with AutoATR System, Shelton, WA, USA) technique was utilized. FTIR spectra were recorded over a wavenumber range from 500 to 4000 cm^−1^, and the resolution was set to 4 cm^−1^. The crystallinity degree of starch powders was analyzed using an X-ray diffractometer (XRD, Empyreen, Malvern Panalytical, Malvern, UK). The starch powders (0.2 g) were recorded via an XRD using Cu-Ka irradiation generated at 45 kV and 40 mA in a theta–theta setup with scanning diffraction angles (2θ) of from 20° to 80° in steps of 3.3°.

### 2.3. Morphology Observation

The modified starch powders or original acetylated distarch phosphate powders were fixed to the metal support and coated with gold via a sputter coater (Ion Sputter E101, Hitachi, Tokyo, Japan). Different modified starch powders were imaged using an S-3000H scanning electron microscope (Hitachi) at 15 kV under low vacuum conditions. Pictures were taken with two different magnifications, 100× and 1000×. Random particles from each modified starch powder were measured on the length of the major axis using the Scion image software (version number 4.0).

### 2.4. Measurement of Water Absorption

A total of 0.2 g of starch powders was added into 4 mL of distilled water and stirred until the hydrogel was formed. The mixture was centrifuged at 5000 rpm for 5 min, unabsorbed water was removed, and then the mixture with the saturated water was weighed. The water absorption of different modified starch powders was calculated using the following equation: water absorption = (Ww/W0) × 100%. Ww was the weight of the saturated hydrogel, and W0 was the initial weight of starch powders.

### 2.5. Viscosity Analysis

The viscosity of modified starch powders or original acetylated distarch phosphate powders was measured using a RheoPlus rheometer (MCR 302e, Anton Paar, Graz, Austria). A total of 0.5 g of starch powders was added into 6 mL of distilled water and stirred until the hydrogel was formed. The hydrogel was rotated at a shear rate of 10 1/s to obtain viscosity, and the measurement was recorded every 10 s with 10 repetitions.

### 2.6. Preliminary In Vivo Anti-Adhesion Testing Procedure

Sprague-Dawley rats, aged 8 weeks and weighing more than 200 g, were used in this study. The experimental procedure was performed according to the Institutional Animal Care and Use Committee (IACUC) of the Master Laboratory Co., Ltd. (Taipei, Taiwan) (IACUC approval no. 22T10-14). The animals were housed in a room at 22 ± 3 °C, relative humidity of 50 ± 20%, and alternating 12 h light-dark cycle. Each animal was provided with food and RO water ad libitum. General anesthesia was achieved by injecting Telazol (50 mg/mL, 4–5 mg/kg) and Balanzine 10% (4–5 mg/kg) into the muscles of the animals. The animals were placed in a supine position, and the abdomens were shaved and prepared using an iodine solution. In each animal, an approximately 5 cm (length of midline) skin incision was made in the abdominopelvic area. The cecal peritoneum was gently abraded repeatedly over a 1 cm × 2 cm area in a standard manner using a dry gauze until visceral peritoneum removal resulted in sub-serosal bleeding and the creation of a homogenous surface of petechial hemorrhages. The parietal peritoneum and inner muscle layer were also sharply dissected in order to create a 1 cm × 2 cm abdominal wall defect. Both injured areas were approximated using a nonabsorbable suture, and three different modified starches or normal saline (shame) were applied to the defect tissues (each rat received only one material). After treatment, the animals were given antibiotics intramuscularly once a day for seven days. Two weeks post-treatment, the animals were sacrificed, and the defect areas were evaluated for adhesion presence.

### 2.7. Histology

Following the sacrifice, the abdominal walls were removed. The specimens were preserved in 10% neutral buffered formalin (NBF) and then trimmed, embedded in paraffin, sectioned, stained with hematoxylin and eosin (H&E) or Masson’s trichrome (MT), and examined microscopically by a veterinary pathologist for adhesive tissue thickness and injury healing. The histological parameters of anastomotic healing are shown in Table 1.

### 2.8. Statistical Methods

The differences in water absorption and viscosity were compared between nonmodified and one kind of salt-modified starch powders or between one kind of salt-modified and another kind of salt-modified starches. The 2-tailed *t*-test was assessed, and a value of *p* < 0.05 was considered significant. For in vivo anti-adhesion testing, data were expressed as a mean ± standard deviation (SD). One-way ANOVA (SPSS, Ver. 22.0) followed by Dunnett’s test was applied for comparison between sham and treatment groups (three different modified starches). *p* < 0.05 was considered significant.

## 3. Results

### 3.1. Different Salt-Modified Starches Demonstrate Different Patterns of Functional Groups

**Figure 1:**
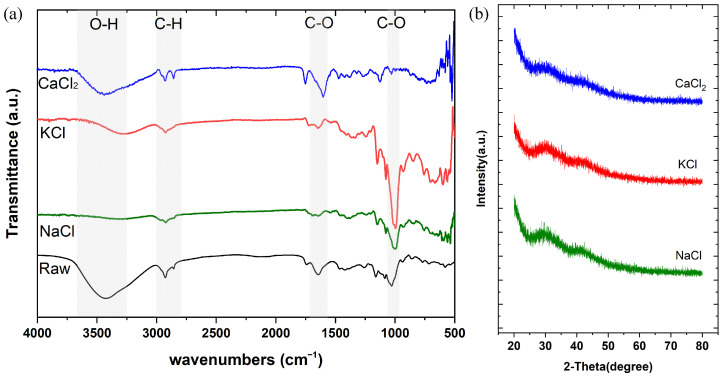
(**a**) Functional group changes in different salt-modified starch powders (CaCl_2_, KCl, or NaCl) and nonmodified acetylated distarch phosphate powders (raw). (**b**) XRD analysis of different salt-modified starch powders displays similar patterns.

In order to confirm whether salt modification changed the functional groups of acetylated distarch phosphate powders, FTIR analysis was performed. After salt modification, the hydroxyl bond was reduced in KCl- and NaCl-modified starch powders (Figure 1a). Although the hydroxyl group in CaCl_2_-modified starch powders could be observed, the transmittance was still less than for raw (acetylated distarch phosphate) starch. The carboxyl group at 1000 cm^−1^ in KCl-modified starch was increased, while the same functional group in CaCl_2_-modified starch was decreased (Figure 1a). When analyzing the crystallinity degree of salt-modified starch powders, the XRD pattern was similar among them (Figure 1b).

### 3.2. Salt-Modified Starches Display Distinct Morphology

**Figure 2:**
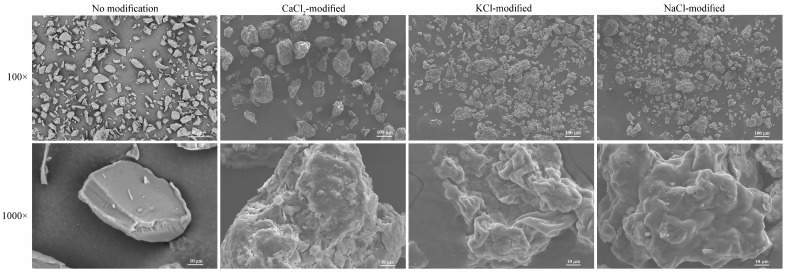
Morphology images of starch powders with or without salt modification captured using scanning electronic microscope. Scale bar shows 100 μm with 100× magnification and 10 μm with 1000× magnification.

After acetylated distarch phosphate powders were modified with different salts, the surface morphology was observed and compared with starch powders without salt modification. Figure 2 shows that when starch powders were not modified with salts, the surface was smooth. When starch powders were modified with salts, the surfaces were irregular and displayed folds regardless of the salt type. The average radius of starch powders modified with CaCl_2_ was 82.1 ± 21.2 μm; with KCl, it was 61.42 ± 23.8 μm, and with NaCl, it was 49.8 ± 25.6 μm. The average radius of acetylated distarch phosphate powders was 62.91 ± 20.1 μm, with the size being closer to KCl-modified starch powders.

### 3.3. Salts Increase Water Absorption

**Figure 3:**
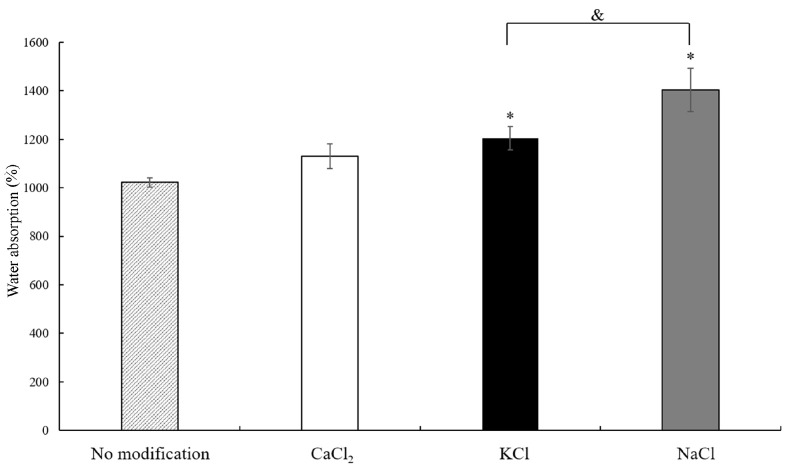
Water absorption of starches is increased when starches are modified with salts. * *p* < 0.05 when comparing water absorption of salt-modified starch powders versus nonmodified starches. ^&^ *p* < 0.05 when comparing water absorption of NaCl-modified starch powders versus KCl-modified starches. Each bar represents average ± standard deviation (SD).

In order to achieve a hemostasis or wound dressing function, starch powders need to be able to absorb water. In Figure 3, it is seen that even when acetylated distarch phosphate powders are not modified by any salts, water absorption can reach 1021.96 ± 19.27%. When starch powders are modified with salts, their water absorption is greatly increased. The water absorption of CaCl_2_-modified starches was 1129.76 ± 51.13%; for KCl-modified starches, it was 1205.31 ± 48.40%, and for NaCl-modified starches, it was 1403.17 ± 88.98%. NaCl-modified starch powders showed the highest water absorption capability.

### 3.4. Salts Dramatically Increase Viscosity

**Figure 4:**
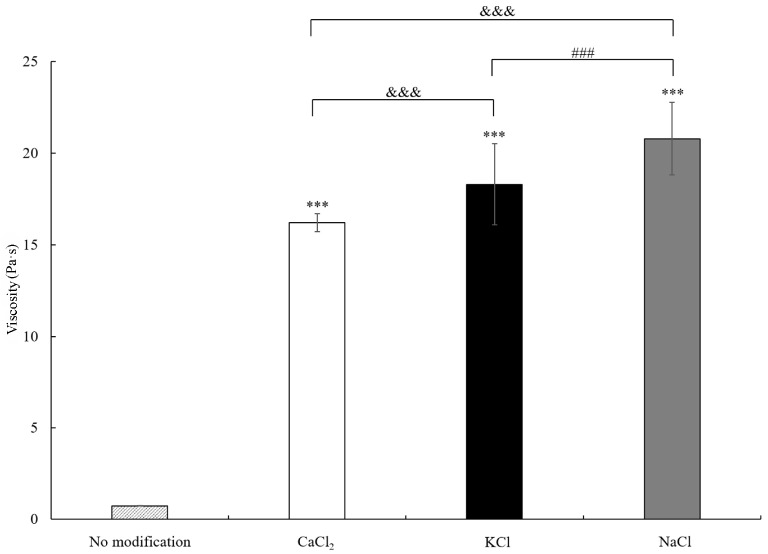
Viscosity is greatly increased when starch powders are modified by salts. *** *p* < 0.001 when comparing viscosity of salt-modified starch hydrogel versus nonmodified starch hydrogel. ^&&&^ *p* < 0.001 when comparing viscosity of CaCl_2_-modified starch hydrogel versus KCl- or NaCl-modified starch hydrogel. ^###^ *p* < 0.001 when comparing viscosity of KCl-modified starch hydrogel versus NaCl-modified starch hydrogel. Each bar represents average ± SD.

Once starch powders absorb water and form a hydrogel, viscosity becomes a critical characteristic for adhesion prevention. Figure 4 displays the viscosity data, and the viscosity of starch powders without salt modification was 0.74 ± 0.01 Pa·s. When salts were added into the starch powders during the modification process, the viscosity was greatly increased. The viscosities of CaCl_2_-, KCl-, and NaCl-modified starch hydrogels were 16.22 ± 0.49, 18.30 ± 2.21, and 20.79 ± 1.99 Pa·s, respectively. The viscosity of NaCl-modified starch hydrogel and its water absorption capability were the highest (Figure 3).

### 3.5. NaCl-Modified Starch Powders Show Better Adhesion Prevention In Vivo

**Figure 5:**
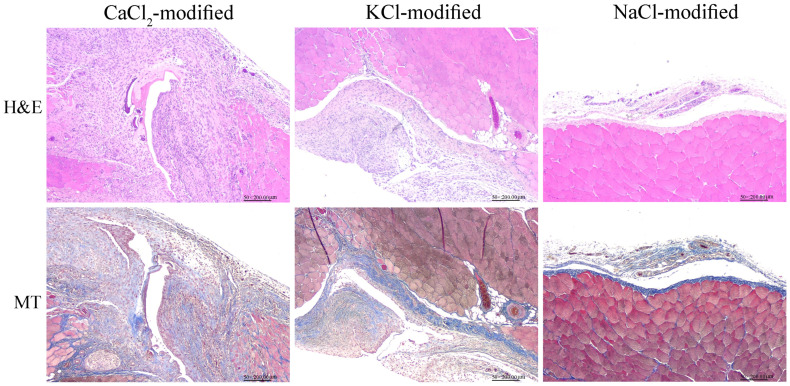
Histology analysis of hematoxylin and eosin (H&E) and Masson’s trichrome (MT) staining following the application of different salt-modified starch powders to the injury site for 2 weeks.

The most direct method to investigate whether salt-modified starch powders could prevent adhesion involved applying starch powders onto the injury sites of animals. Histological results demonstrated an unorganized injury site following the application of CaCl_2_-modified starch powders but more organized, recovered injury sites when NaCl-modified starch powders were applied (Figure 5). In addition, NaCl-modified starch powders caused the thinnest adhesive tissue (Table 2) and also the lowest injury healing score (Table 3), suggesting that NaCl-modified starch powder had a better effect on adhesion prevention.

## 4. Discussion

Water absorption capacity is a critical factor if starches are applied as hemostasis, wound dressing, or anti-adhesion. The water absorption of native potato starch was shown to be 270.22% [31]. The acetylated distarch phosphate powders used in this study exhibited higher water absorption, suggesting that the acetylation of potato starch leads to improved water absorption ability. The acetylated distarch phosphate is obtained by heating potato starch in acetic acid, resulting in the replacement of some hydroxyl groups on anhydroglucose units with acetyl groups [32]. During the acetylation process, the hydrogen bonds between starch molecules weaken, causing starch molecules to bind with water and increasing water absorption [33]. Therefore, the water absorption of acetylated distarch phosphate powders increased to 1021% (Figure 3), which was higher than that of native potato starch [31]. Even though the water absorption capability of acetylated distarch phosphate powder is high, it might not be sufficient to form high-viscosity hydrogels that can be applied in wound cavities as a dressing or as an anti-adhesion agent, providing a physical barrier between two body surfaces. Therefore, different salts were added to investigate the potential biomedical function of acetylated distarch phosphate powder, and overall, they improved the water absorption abilities of these powders (Figure 3). Emulsification was used here to incorporate salts into acetylated distarch phosphate powder through physical modification. Because both acetylated distarch phosphate powders and salts could dissolve in water, both molecules could tightly aggregate when being poured into the oil phase. After emulsification, the surface morphology of salt-modified acetylated distarch phosphate powders became irregular. One possible reason was that an irregular surface resulted in a larger surface area of salt-modified starch powders and, subsequently, in the retention of more water molecules. Additionally, it has been shown that an increase in water absorption capacity is related to an increase in the surface area of horse chestnut starch, water chestnut starch, lotus stem starch, and corn starch [34,35]. Another explanation may be that the presence of ions in salts disrupts parts of the hydrogen bonds between acetylated distarch phosphate molecules, resulting in higher water absorption.

**Figure 6:**
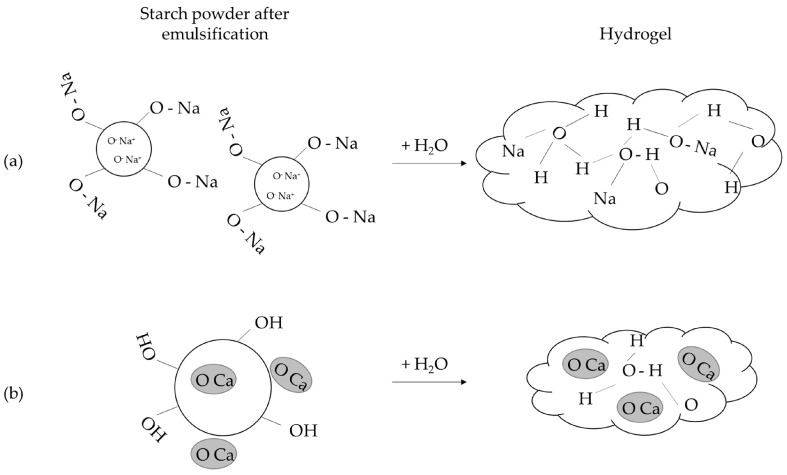
The model illustrates the potential mechanism of salt-modified starch powder in improving water absorption and viscosity. (**a**) Na^+^ replaces the hydrogen ion on the surface and in the middle of starch after emulsification with NaCl. Because the particle size is smaller, more starch particles can be contained, resulting in a larger surface area and more water molecules. Therefore, tighter networks of the hydrogel are formed and display higher viscosity. (**b**) Ca^2^+ may form additional crosslinks with the carboxylate groups (the grey circle) after emulsification, disrupting the intermolecular and intramolecular structure and leading to a lower viscosity of the hydrogel.

This poses the question of why NaCl-modified starch powders display higher water absorption than CaCl_2_- or KCl-modified starch powders. The first observation was changes in some functional groups (Figure 1a). The hydroxyl group in NaCl-modified starch was greatly reduced when compared with the other three starch powders. It has been hypothesized that starch is a weak ion exchanger [36]; thus, starch-OH can form starch-O-Na covalent bonds. In contrast, CaCl_2_-modified starch powder displayed decreased carboxylate bonds, while the hydroxyl group showed a similar pattern to the raw starch (Figure 1a). Bivalent cations have been shown to coordinate with the carboxylate groups on the hydrogel. This coordination could induce additional crosslinks, resulting in a decrease in the water penetrating into the networks of the hydrogel and lower water absorption [37,38]. Therefore, Ca^2+^ prefers to form crosslinks with the carboxylate groups rather than to form starch-O-Ca covalent bonds (Figure 6b). Accordingly, we hypothesized that the surface of NaCl-modified starch displayed starch-O-Na covalent bonds and that Na^+^ could be easily replaced by H^+^ following the addition of water (Figure 6a).

The second observation was that the powder size of NaCl-modified starch was smaller than CaCl_2_- or KCl-modified starch powders; thus, the total surface area of NaCl-modified starch is bigger under the same gram number, resulting in higher water absorption. A previous study showed that the water absorbency of a wheat straw cellulose-based semi-interpenetrating polymer networks hydrogel in different salt solutions follows the swelling ratio order Na^+^ > K^+^ > Mg^2+^ > Ca^2+^ and suggested that this was mainly caused by the ion size [39]. A smaller-sized ion, such as Na^+^, can more easily penetrate the networks of the starch hydrogel and attract more water molecules (Figure 6a). 

Clinically, an anti-adhesive hydrogel with high osmotic pressure and high viscosity can block or minimize tissue growth to prevent postoperative adhesion [40]. According to our results on water absorption, NaCl-modified starch hydrogel also displayed the highest viscosity (Figure 4). When acetylated distarch phosphate powder was not modified with salts, the viscosity was much lower than that of the salt-modified starch hydrogel. Previous studies have demonstrated a decrease in viscosity after the acetylation of potato starch, oat starch, and canna starch [41,42,43]. The possible reason for this might be the weakening starch granule resulting from the disruption of the inter- and intramolecular bonds of acetylation or decreased bonding with water molecules because of the hydrophobicity of acetyl groups [43]. Since the water absorption of acetylated distarch phosphate powder was higher than that of the native potato starches, the disruption of the inter- and intramolecular bonds could have been the cause of its low viscosity here. For NaCl-modified starch hydrogel, Na^+^ could penetrate the hydrogel more easily and attract more water molecules that might form tight networks between the starch and water (Figure 6a). In contrast, CaCl_2_-modified starch hydrogel might form crosslinks between Ca^2+^ and the carboxylate groups of the starch. These additional crosslinks might result in weakened bonding with water molecules and reduced viscosity (Figure 6b). The viscosity property of salt-modified starch hydrogel corresponded to the result of in vivo adhesion analysis. NaCl-modified starch hydrogel showed higher viscosity, and its adhesion prevention ability was better. Other studies have also demonstrated that higher hydrogel viscosity and better adhesion prevention performance are correlated [44,45].

In this study, salt-modified starch powder was shown to improve water absorption and viscosity, and the preliminary in vivo result displayed an anti-adhesion function. Although water absorption capacity has been shown to be a critical factor for starch-based powders to act in hemostasis, other factors can also contribute to this pathway. Calcium ions, for example, play an important role in the tight regulation of the coagulation cascade, which is crucial in the maintenance of hemostasis [46,47]. It was interesting to investigate whether CaCl_2_-modified starch powders displayed better hemostatic function despite their water absorption ability being lower than that of NaCl-modified starch powders. It was also interesting to investigate whether salt-modified starch powder can be applied as a wound dressing. Because wound healing is a complex process, simply having good water absorption capacity and high viscosity is not sufficient. Other materials may need to be added into salt-modified starch powders in order for them to exhibit suitable mechanical properties, antimicrobial activities, an ideal water/exudate ratio, and good antioxidant and anti-inflammatory characteristics, as these are required for wound dressings [48,49]. However, the results of this study demonstrated that the water absorption capacity of acetylated distarch phosphate powders could be improved with the addition of salt, regardless of the kind. In addition, the results also demonstrated that the high water absorption and viscosity properties of salt-modified starch powders lead to better adhesion performance in vivo.

## 5. Conclusions

The current study demonstrates that the water absorption ability and viscosity of acetylated distarch phosphate potato starch powders can be greatly improved by the addition of salts. Among the three different salts tested here, NaCl-modified starch powders showed the highest water absorption and viscosity. An in vivo anti-adhesion test also demonstrated that NaCl-modified starch powders could effectively prevent adhesion at the injury site. These results suggest that NaCl-modified acetylated distarch phosphate potato starch powders could potentially act as anti-adhesive materials.

## Figures and Tables

**Table 1:** The histological parameters of anastomotic healing and grading system. Lower scores represent better healing.

Parameter	Description	Grading
Inflammation ^1^	Presence of inflammatory cells at the anastomosis	0 = no; 1 = <10%; 2 = 10–39%; 3 = 40–79%; 4 = 80–100%
Injury recovery ^1^	Assess the tissue and mucosal injury has recovered in the intestinal serosa and the peritoneum	0 = recovery; 1 = no recovery

^1^ The anastomotic healing score was calculated as the sum of scores of these parameters.

**Table 2:** Adhesion thickness assessment of injury site with different salt-modified starch powders.

	Group ^b^		
Measurements ^a^	CaCl_2_	KCl	NaCl
Adhesive tissue thickness (μm)	1018.37 ± 311.34 *	386.78 ± 150.65	185.59 ± 31.24

^a^ Values represent mean ± standard deviation. ^b^ Significant differences (*: *p* < 0.05) between CaCl_2_-modified group and other treated groups using Dunnett’s one-way ANOVA.

**Table 3:** Anastomotic healing score in injury site with different salt-modified starch powders (for scoring, see Table 1).

	Group ^b^		
Measurements ^a^	CaCl_2_	KCl	NaCl
Inflammation	2.67 ± 0.58	1.33 ± 0.58	0.33 ± 0.58
Injury recovery	0.67 ± 0.58	0.33 ± 0.58	0.00 ± 0.00
Injury healing score	3.33 ± 0.58 *	1.67 ± 0.58 *	0.33 ± 0.58

^a^ Values represent mean ± standard deviation. ^b^ Significant differences (*: *p* < 0.05) between sham and other treated groups using Dunnett’s one-way ANOVA.

## Data Availability

The data presented in this study are available on request from the corresponding authors.
